# One anastomosis gastric bypass with fundoplication of remnant stomach for weight regain prevention: Case report

**DOI:** 10.1016/j.ijscr.2022.107431

**Published:** 2022-07-20

**Authors:** O. Ospanov, K. Nadirov, V. Koikov, N. Zharov

**Affiliations:** Department of Surgical Disease and Bariatric Surgery, Astana Medical University, Nur-Sultan, Kazakhstan

**Keywords:** OAGB, one anastomosis gastric bypass, SCARE, Surgical CAse Report, BMI, body mass index, Case report, Bariatric surgery, One anastomosis gastric bypass, Weight regain prevention, Fundoplication, FundoRingOAGB

## Abstract

**Introduction:**

Weight regain is a serious issue after bariatric surgery. Using banding for one anastomotic gastric bypass prevents weight regain, but carries the risk of band erosion. The combination of bariatric procedures and fundoplication for patients without hiatal hernia probably prevents weight regain. However, no studies are demonstrating this.

**Presentation of case:**

A 38-year-old woman who underwent laparoscopic one anastomosis gastric bypass with fundoplication of remnant stomach maintained a stable body weight after 5 years.

**Discussion:**

Owing to the complications associated with the conventional methods, the combination treatment has several advantages such as prevention leak of suture line in the gastric pouch prevention.

**Conclusion:**

The combination treatment could successfully prevent weight regain and thus, will be helpful in the better management of obesity.

## Highlights

• The combination of bariatric treatment prevents weight regain, bile and acid reflux.
• Complete coverage of weak site by the fundoplication wrap for better safety
• Advantages of FundoRingOAGB over Nissen fundoplication can be listed.

## 1 Introduction

Weight regain after bariatric surgery is a great challenge [1]. Various mechanical implants involving adjustable bands and rings such as “FobiRing” are being used to mitigate weight regain. However, the application of these foreign materials can cause complications like the erosion of the stomach wall [2]. Hence, surgeons avoid the use of various mechanical implants.

The novel concept of banding the esophageal-gastric junction with the use of autologous banding tissue seems to be important, especially for one anastomotic gastric bypass, the benefits of which are offset by the likelihood of biliary reflux into the esophagus [3]. Unfortunately, there are no studies investigating the effect of combining the two procedures on the prevention of weight regain after bariatric procedures.

Herein, we present a surgical technique and report a case of weight regain prevention using a combination of methods. We carried out laparoscopic one anastomosis gastric bypass (OAGB) with fundoplication of the remnant stomach in a patient without hiatal hernia with a 5-years follow-up. This report is in line with the Surgical CAse Report (SCARE) criteria [4].

## 2 Presentation of case

### 2.1 Patient

A 39-year-old woman with a history of laparoscopic mini-gastric bypass with a fundoplication for morbid obesity [body mass index (BMI) 41.4 kg/m^2^] was followed-up at our bariatric clinic after 5 years of the surgery. There was no history of medications or smoking. The family history involved obesity in both parents.

During the operation, a hiatal hernia was not observed. However, due to the complaint of heartburn, a one-stage fundoplication was performed.

### 2.2 Surgical technique

The procedure was performed by a certified bariatric surgeon with more than 10 years of experience in gastric bypass surgery.

**Fig. 1:**
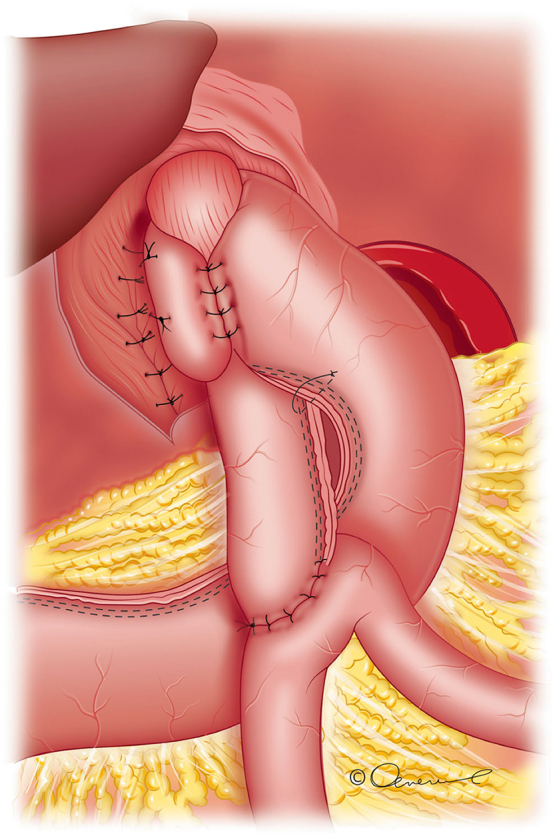
Schema of the performed surgical procedure.

The overview of the surgical procedure is shown schematically in Fig. 1.

**Fig. 2:**
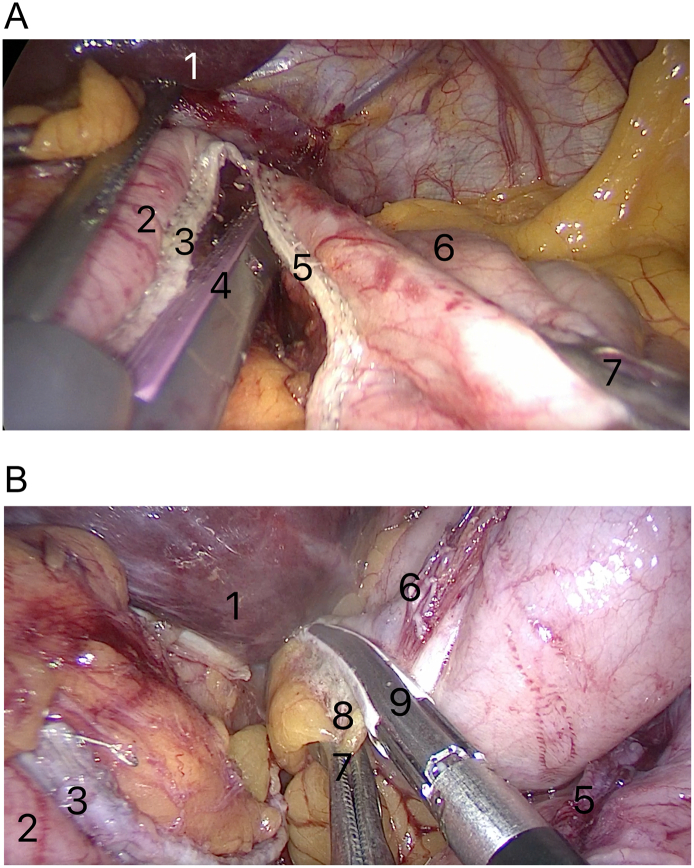
The stomach division into two parts: the gastric pouch (left) and the excluded part of the stomach (right) using stapler EndoGIA-60 [A]. Gastrosplenic ligament divided using LigaSure [B]. Marked on the illustrations: 1—hepar; 2—gastric pouch; 3—staple line of gastric pouch; 4—EndoGIA-60; 5—staple line of the excluded part of the stomach; 6—fundus of the excluded part of the stomach; 7—grasper; 8—gastrosplenic ligament; 9—LigaSure instrument.

In the beginning, we laparoscopically performed the standard gastric pouch for one anastomosis gastric bypass [5] (Fig. 2A). The excluded part of the stomach was additionally mobilized by dissecting the short gastric vessels connecting the gastrosplenic ligament (Fig. 2B).

**Fig. 3:**
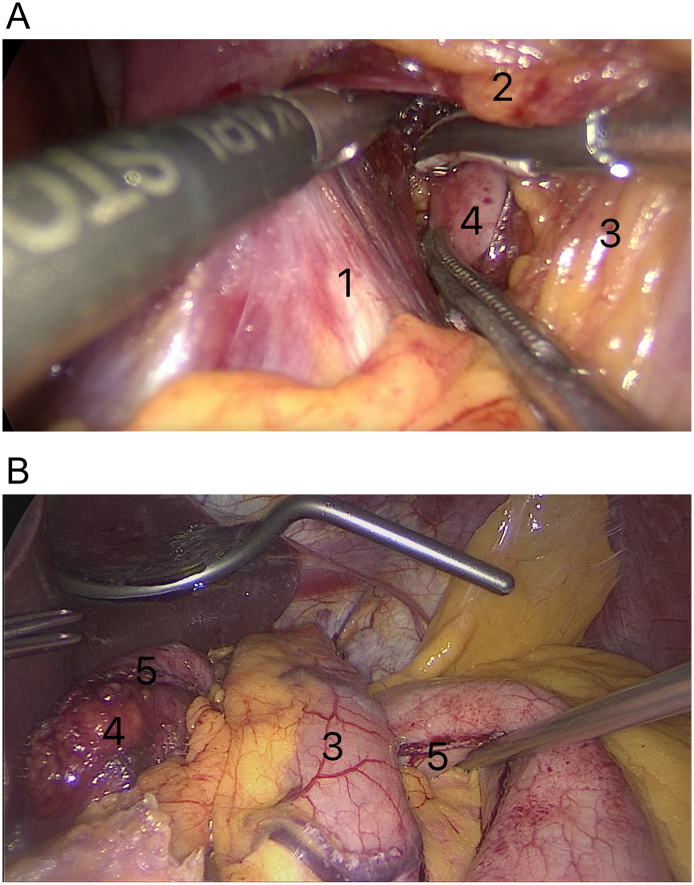
Creation retrogastroesophageal canal [A]. Complete mobilize the fundus of the excluded part of the stomach movement through retrogastroesophageal canal (“Shoeshine” maneuver) [B]. Marked on the illustrations: 1—diaphragmatic crura; 2—esophagus; 3—gastric pouch; 4—fundus of the excluded part of the stomach in retrogastroesophageal window; 5—stapler suture line of the excluded part of the stomach.

The dissection of short gastric vessels increased the mobilization of the fundus of the stomach. This leads to its free movement through the retrogastroesophageal canal under the intra-abdominal segment of the esophagus (3/4 part of wrap) and under the upper part of the gastric pouch (1/4 part of wrap) (Fig. 3A, B).

**Fig. 4:**
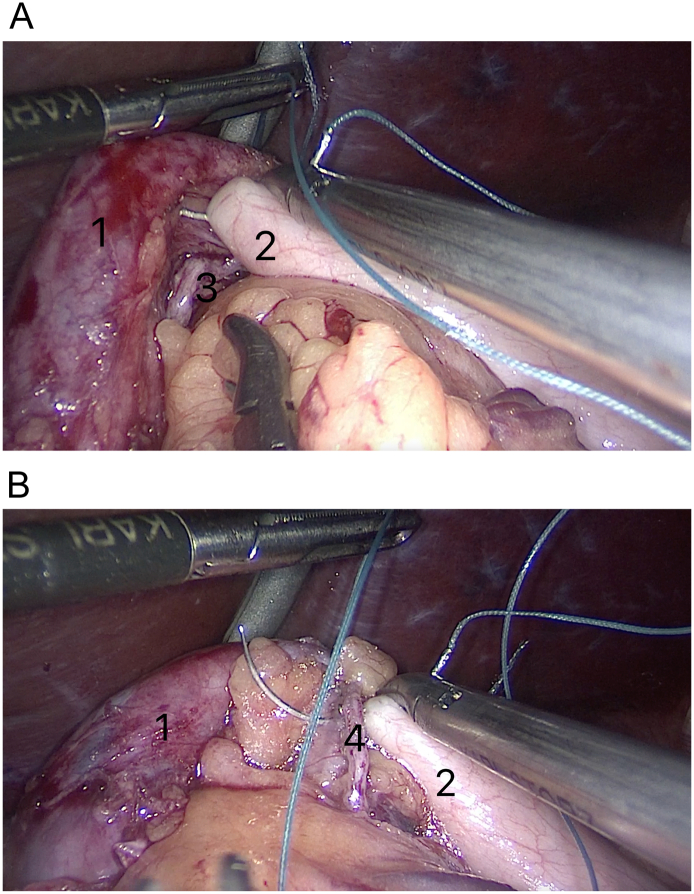
Stitching the anterior and posterior parts of the fundoplication wrap of the excluded part of the stomach [a]. Simultaneously wrap stitched through stapler suture line of gastric pouch at the lower part of the wrap [b]. Marked on the illustrations: 1—posterior part of the fundoplication wrap of the excluded part of the stomach; 2—anterior part of the fundoplication wrap of the excluded part of the stomach; 3—stapler suture line of the excluded part of the stomach; 4—stapler suture line of gastric pouch.

As in standard Nissen fundoplication, we sewed the anterior and posterior parts of the fundoplication wrap with non-absorbable Etibond 2/0 sutures. However, unlike the Nissen method, we used a staple suture line of stomach during the procedure. To improve the reliability of the seam, the staple suture line of the gastric pouch was covered and nestled inside the anterior part of the wrap (Fig. 4a).

Besides, unlike the standard Nissen fundoplication:

a) for the creation of the posterior part of the wrap we captured the widely mobilized fundus in the upper part of the staple line of the remnant stomach and was passed behind the intra-abdominal segment of the esophagus from left to right. This step was performed after the left and right crural dissection and opening of the gastrohepatic ligament beginning at the pars flaccida.
b) the upper edge of the stapler suture line of the gastric pouch was simultaneously stitched to the lower part of the wrap between the anterior and posterior parts to prevent slipping of the wrap and for the correct functioning of the anti-reflux mechanism (Fig. 4b).

Moreover, unlike the Nissen method, we stitched the edges of the wrap at the 1 o'clock position on the anterior aspect of the esophagus (not at 10 o'clock as in the Nissen method) [6]. This 1 o'clock position was necessary for the correspondence of the stitching point of the anterior and posterior parts of the fundus of the remnant stomach to the point of the stapler line on the gastric pouch. Moreover, this prevented malformation of the fundoplication. The remaining sutures were placed sequentially to cover a total of 3–5 cm of the intra-abdominal segment of the esophagus.

The anterior part of fundoplication was extended downwards, along the stapled suture line of the gastric pouch, for approximately 2 cm for the creation of additional partial fundoplication. Thus, we created an upper: total fundoplication around the intra-abdominal segment of the esophagus and lower: a partial fundoplication to the left of the stapled line of the gastric pouch. For calibration, we used a gastric bougie of size 32 Fr.

**Fig. 5:**
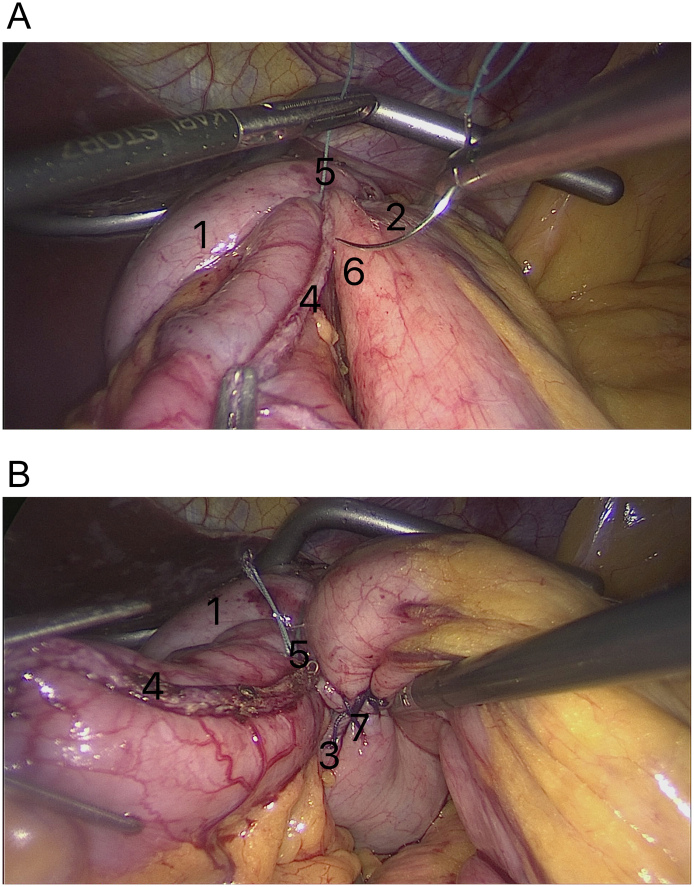
After circularly fundoplication we created partial fundoplication with the anterior stitching of the edges of the wrap also at 1 o'clock [A]. And performed the additional posterior stitching at 3 o'clock as a “live ring” [B]. Marked on the illustrations: 1—posterior (right) part of the fundoplication wrap of the excluded part of the stomach; 2—anterior (left) part of the fundoplication wrap of the excluded part of the stomach; 3—stapler suture line of the excluded part of the stomach; 4—stapler suture line of gastric pouch; 5—suture between anterior (left) part of the excluded part of the stomach and stapler suture line of gastric pouch for created partial fundoplication; 6—place of anterior (left) part of the excluded part of the stomach for completed partial fundoplication; 7—the additional posterior stitching between the edge of partial fundoplication and stapler suture line of posterior part of the fundoplication wrap of the excluded part of the stomach.

The next difference is the additional suturing between the anterior and posterior parts of the fundoplication wrap at 3 o'clock to form a “live ring” (“FundoRing”) (Fig. 5A, B).

Subsequently, the routine surgical technique of OAGB was followed. A jejunal loop measuring 200 cm from the ligament of Treitz was anastomosed to the gastric pouch. The two-layer hand-sewn 2 cm gastrojejunostomy was created by using absorbable sutures (Vicryl 2/0) (Ethicon).

The anastomosis was tested using methylene blue injection, and the port sites were subsequently closed using the standard closure techniques.

### 2.3 Postoperative management

The patient received an intravenous infusion with antibiotics before the procedure and for 1 day after the procedure. The proton pump inhibitor was administered over 24 h and then taken orally for 6 weeks. The patient started a clear liquid diet on the 1st postoperative day. The patient was discharged on the 3rd postoperative day with adequate diet tolerance.

### 2.4 Evaluation of the outcome during follow-up

**Fig. 6:**
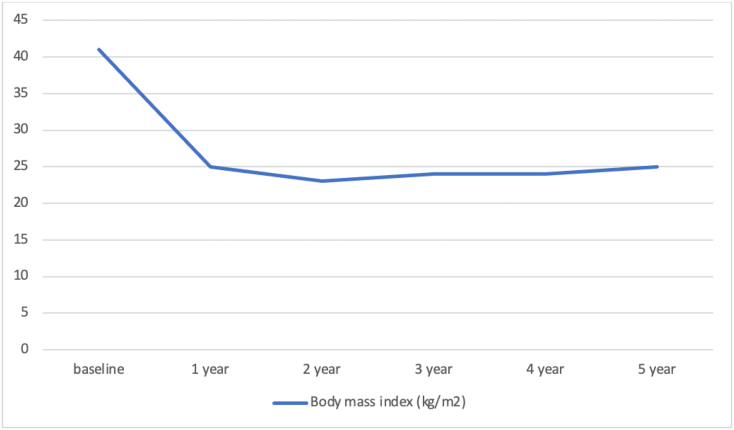
Follow-up analysis of body mass index within five-years.

The follow-up was carried out every year up to 5 years after the surgery. We evaluated the body mass index before the surgery and during the follow-up (Fig. 6). The BMI was 41.4 kg/m^2^ before surgery. After surgery, the BMI were 25.6 kg/m^2^, 23.1 kg/m^2^, 24.1 kg/m^2^, 24.3 kg/m^2^ and 25.4 kg/m^2^ after the first year to the fifth year respectively. After 5 years, the patient expressed his satisfaction with the results of the procedure.

## 3 Discussion

Various bariatric interventions such as hypocaloric diet therapy and gastric bypass are being used for the management of obesity. Compared to the hypocaloric diet therapy, gastric bypass resulted in greater body mass loss and greater metabolic syndrome resolution, but none of these interventions have an anti-reflux component, such as a fundoplication [7].

Moreover, the gastric bypass has a high incidence of complications such as dumping syndrome, reflux esophagitis, and recurrence of obesity. Therefore, there has been an increase in the popularity of combined bariatric procedures and fundoplication for revisional bariatric surgery.

We have proposed a classification of combination procedures wherein we have presented a novel combined method called “FundoRingOAGB” [3]. Furthermore, we are one of the first to suggest this method as a primary operation to prevent weight regain. This case report describes the surgical technique and reports that the application of fundoplication of the remnant stomach despite the absence of hiatal hernia, in combination with gastric bypass, leads to the prevention of obesity recurrence.

For the first time, we would like to highlight the advantages of FundoRingOAGB over the conventional Nissen fundoplication:

1. Complete anatomical separation of the fundus of the stomach by dissecting the short gastric vessels of the fundus facilitated tension-free and wide fundoplication around the intra-abdominal segment of the esophagus and upper part of the gastric pouch.
2. Complete coverage of the area that is susceptible to leak of suture line in the gastric pouch in the upper part increases the safety.
3. Demonstrating the possibility of extension of fundoplication downwards along the stapled line of the gastric pouch as an additional partial Fundo(gastro)plication.
4. It is possible to correct the tension of the wrap using calibration. The covering of the suture line of the gastric pouch by the lower surfaces of the fundoplication wrap according to the type of a living ring.
5. A wide and calibrated wrap around the gastric pouch prevents its dilatation and gaining of weight in the late postoperative period.
6. The combination of one anastomosis gastric bypass with fundoplication of the remnant stomach can be applied not only to prevent weight regain but also for the prevention of biliary reflux and acid reflux.

The preliminary data of this clinical case showing promising outcomes led us to the initiation of a randomized controlled trial to further validate the results [8]. We are optimistic about the validation of our 5-year-follow up result in our randomized trial with a large number of patients in the sample.

## 4 Conclusion

The combination of one anastomosis gastric bypass with fundoplication of the remnant stomach is effective and could be used for better management of obesity.

## Consent for publication

Written informed consent was obtained from the patient and her family for the publication of this case report and the accompanying images. A copy of the written consent is available for review by the Editor in Chief of this journal upon request.

## Provenance and peer review

Not commissioned, externally peer-reviewed.

## Ethical approval

Not applicable.

## Funding

This research did not receive any specific grant from funding agencies in the public, commercial, or not-for-profit sectors.

## Guarantor

Oral Ospanov.

## Research registration number

ClinicalTrials.gov identifier: NCT04834635.

## CRediT authorship contribution statement

Oral Ospanov is the corresponding author and conceptualized of treatment and the manuscript.

Kamalzan Nadirov, Vitaly Koikov, and Nurlan Zharov contributed to play important role to data collection, data analysis, interpretation, and writing the paper. Oral Ospanov, Kamalzan Nadirov, Vitaly Koikov, and Nurlan Zharov approved the final manuscript.

## Declaration of competing interest

All authors declare no conflicts of interest.
